# Sirtuin 5 inhibits mitochondrial metabolism in liver cancer cells and promotes apoptosis by mediating the desuccinylation of CS

**DOI:** 10.3389/fimmu.2025.1560989

**Published:** 2025-06-10

**Authors:** Huimin Cao, Dongsheng Wei, Han Li, Mei Zhao, Yixin Ma, Liang Kong, Guoyuan Sui, Lianqun Jia

**Affiliations:** ^1^ Key Laboratory of Ministry of Education for Traditional Chinese Medicine (TCM) Viscera-State Theory and Applications, Liaoning University Of Traditional Chinese Medicine, Shenyang, China; ^2^ Department of Life Sciences, Beijing University of Chinese Medicine, Beijing, China; ^3^ College of Pharmacy, Liaoning University of Traditional Chinese Medicine, Dalian, China

**Keywords:** citrate synthase, hepatocellular carcinoma, PTMs (post-translational modifications), succinylation, mitochondrial metabolism, apoptosis

## Abstract

**Background:**

Citrate synthase (CS) is a key rate-limiting enzyme in the tricarboxylic acid (TCA) cycle and plays a crucial role in cancer progression. However, the mechanism by which CS promotes liver cancer growth remains unclear. The aim of this study is to elucidate the role of CS and its post-translational modifications (PTMs) in the initiation and progression of hepatocellular carcinoma (HCC).

**Methods:**

Liquid chromatography-tandem mass spectrometry (LC-MS/MS) was used to detect protein lysine succinylation in human liver cancer and adjacent non-cancerous tissues. A HCC model was established in male C57BL/6 mice through intraperitoneal injection of DEN. The expression of SIRT5 and CS in HCC mice was assessed by RT-qPCR, immunohistochemistry, and Western blotting. HepG2 cells were cultured, and co-immunoprecipitation (Co-IP) was performed to evaluate the interaction between SIRT5 and CS. Western blotting was used to measure the succinylation levels of CS. In addition, Mito-Tracker Red CMXRos staining, reactive oxygen species (ROS) measurement, ATP level assay, EdU cell proliferation assay, colony formation assay, TUNEL staining, and flow cytometry were used to investigate the effects of CS succinylation and desuccinylation on mitochondrial function and cell proliferation in hepatocellular carcinoma cells.

**Results:**

A total of 358 differentially modified proteins were identified in human liver cancer tissues. These differentially modified proteins were primarily enriched in the mitochondria, and CS exhibited high levels of succinylation in HCC tissues. In mouse liver cancer tissues, SIRT5 expression was reduced while CS expression was increased. Furthermore, SIRT5 was found to interact with CS, mediating the de-succinylation of CS at the lysine 375 site. Additionally, succinylation at the K375 site of CS was shown to enhance mitochondrial activity and ATP content in HepG2 cells, while reducing intracellular ROS levels and promoting cell proliferation. In contrast, de-succinylation of CS at the K375 site significantly impaired mitochondrial function and ATP levels, increased ROS levels, and induced apoptosis in HepG2 cells.

**Conclusion:**

Succinylation of CS is crucial for maintaining mitochondrial function and promoting cell proliferation in liver cancer cells. Targeting SIRT5-mediated de-succinylation of CS may represent a promising therapeutic strategy for the treatment of hepatocellular carcinoma.

## Introduction

Hepatic malignancy remains a global health threat, and according to the latest data from the International Agency for Research on Cancer (IARC) 2024 of the World Health Organization, which reveals that there will be 9,740,000 cancer deaths globally in 2022, of which 760,000 deaths from hepatocellular carcinoma will be the highest number (1). HCC characterized by late-stage diagnosis and poor treatment outcomes (2). In recent years, beyond classical genetic alterations and signaling pathways, metabolic reprogramming has emerged as a key driver of HCC initiation and progression (3, 4). Tumor cells adopt adaptive metabolic strategies such as reprogramming of the tricarboxylic acid (TCA) cycle, enhanced lipid biosynthesis, and redox modulation to meet their proliferative demands (5, 6). These metabolic adaptations significantly affect tumor cell survival and invasiveness. Therefore, investigating the regulatory mechanisms of key metabolic enzymes in HCC is essential for uncovering tumor pathogenesis and developing novel therapeutic strategies.

TCA cycle, also known as the citric acid cycle, serves as a central hub for the metabolism of three major nutrients: proteins, lipids, and glucose (7). It plays a crucial role in tumor metabolic reprogramming (8, 9). Citrate synthase (CS) is the rate-limiting enzyme of the TCA cycle and catalyzes the first step of the cycle (10). CS expression is elevated in hepatocytes, and its enzyme activity is aberrant (11); however, the post-translational modifications (PTM) of CS and its regulatory role in HCC development remain unclear. Protein succinylation is a recently discovered post-translational modification, where the succinyl group from a donor is covalently attached to a lysine residue through enzymatic or non-enzymatic processes (12). Lysine succinylation is widely present in both eukaryotic and prokaryotic cells. It plays a regulatory role in various pathways, including the tricarboxylic acid cycle, amino acid metabolism, and fatty acid metabolism. This modification is closely associated with diseases such as neurodegenerative disorders (13), inflammation, metabolic diseases (14), and cancer (15). SIRT5 belongs to the sirtuin family of NAD+-dependent deacetylases and is an important metabolic regulator (16). It primarily removes succinyl, malonyl, and glutaryl groups from lysine residues in mitochondria and peroxisomes (17). SIRT5 has dual roles in promoting or suppressing tumors (18), with its expression being significantly downregulated in liver cancer tissues (19). Recent studies have shown that the role of SIRT5 in the development of renal cell carcinoma, breast cancer, and hepatocellular carcinoma depends on its deacetylase activity (20–22). Research has also found that SIRT5 interacts with CS, and SIRT5 removes succinyl groups from CS at the evolutionarily conserved residues K393 and K395, promoting the proliferation and migration of colon cancer cells (23). Whether SIRT5 regulates CS through its de-succinylation activity to exert its suppressive effect in hepatocellular carcinoma remains unclear.

In this study, a quantitative proteomic analysis of lysine succinylation was conducted on cancerous and adjacent non-cancerous tissues from HCC patients. We discovered the accumulation of lysine succinylation in human liver cancer tissues, with a marked change in the succinylation modification of CS. CS was found to be a substrate of SIRT5, and SIRT5 de-succinylates CS at lysine K370. The high succinylation of CS promoted the proliferation and migration of liver cancer cells. Our findings reveal a novel PTM of CS and provide initial insights into the impact of CS succinylation on hepatocellular growth and migration. These results also suggest potential therapeutic strategies for intervening in tumors by modulating the interaction between CS and SIRT5.

## Materials and methods

### Cell lines and clinical samples

The HepG2 hepatocellular carcinoma cell line was purchased from Wuhan PunoSci Biotechnology Co., Ltd. Cells were cultured in MEM (NEAA) supplemented with 10% fetal bovine serum and 1% penicillin/streptomycin. All cell lines were authenticated by Short Tandem Repeat (STR) analysis and tested for mycoplasma contamination.

Five patients diagnosed with liver cancer were enrolled from Shenyang Chest Hospital. Tumor tissue samples and corresponding adjacent tissue samples (approximately 2 cm from the tumor margin) were collected and stored in liquid nitrogen for further analysis. All participants provided written informed consent to undergo clinical examinations and sample collection. The study protocol was approved by the institutional review board (KYXM-2023-001-02).

### 4D-label free proteomics analysis of peptide succinylation

For 4D-label-free proteomics analysis of peptide succinylation, proteins were extracted using urea-based lysis, followed by reduction with DTT, alkylation with iodoacetamide, and trypsin digestion. Peptides were enriched using anti-succinyl-lysine antibody-conjugated resin (PTM Biolabs, Hangzhou, China) and eluted with 0.1% TFA. After desalting and drying, peptides were analyzed on an EASY-nLC 1000 UPLC system coupled to a Q Exactive Plus mass spectrometer (Thermo Fisher). A data-dependent acquisition mode was used, with a full MS scan (m/z 350–1800) at 70,000 resolution, followed by MS/MS scans of the top 20 ions. MS/MS data were analyzed using MaxQuant (v1.6.6.0) against the Homo sapiens database. Carbamidomethylation (C) was set as a fixed modification, while oxidation (M), acetyl (N-term), and succinylation (K) were variable. Label-free quantification (LFQ) was performed with an FDR of 1% for both PSM and protein levels, and only succinylated peptides with localization probability >0.75 were retained.

### Bioinformatics analysis

The GSE84402 dataset was derived from Gene Expression Omnibus (GEO) and included 18 normal samples and 18 HCC samples. R software and T test were used to analyze the differential expression of CS and sirtin(SIRT)5 in 18 normal and 18 HCC patients.

### Animal studies

The animal experimental protocol in this study was approved by the Animal Ethics Committee of Liaoning University of Traditional Chinese Medicine in accordance with the “Guide for the Care and Use of Laboratory Animals” (National Research Council) (Ethics ID: 21000042022042). Twenty SPF-grade male C57BL/6 mice, aged 3 weeks, with an average body weight of (10 ± 2) g, were purchased from Beijing Vital River Laboratory Animal Technology Co., Ltd. (Animal License No. SCXK (Beijing) 2016-0006). The mice were housed in the Animal Facility of Liaoning University of Chinese Medicine, with a relative humidity of 50%, room temperature maintained at 22°C, natural lighting, and free access to water and food. The mice were randomly divided into two groups: the NC group and the HCC group, with 8 mice in each group. Mice in the HCC group were intraperitoneally injected with DEN (50 mg/kg) (DEN (#N0725)Sigma Aldrich, St.Louis, MO)once a week for 8 consecutive weeks to establish the HCC model. After the model was successfully established, the mice were euthanized, and their livers were harvested, weighed, and subjected to RT-qPCR, immunohistochemistry, and Western blotting analyses.

### RT-qPCR

Total RNA was extracted from liver cancer tissues using Trizol reagent(CW0580, CoWin Biotech Co.,Ltd,Jiangsu,China). The RNA was then reverse transcribed into cDNA using the HiFiScript gDNA Removal cDNA Synthesis Kit( CW2582,CoWin Biotech Co.,Ltd,Jiangsu,China). Quantitative PCR (qPCR) was performed using the UltraSYBR Mixture (Low ROX) Kit(Cw2601,CoWin Biotech Co.,Ltd,Jiangsu,China). The reaction conditions were set as follows: 95°C for 10 minutes, followed by 40 cycles of 95°C for 15 seconds and 60°C for 1 minute. Primers were synthesized by Shanghai Shenggong Bioengineering Co., Ltd., and the primer sequences for CS, sirtuin (SIRT) 5, and GAPDH are provided below.CS, forward, 5′-CTCATCCTGCCTCGTCCTTG-3′ and reverse, 5′-GCCACCGTACATCATGTCCAC-3′;sirtuin (SIRT)5, forward, 5′-GATTCATTTCCCAGTTGTGTTGT-3′ and reverse, 5′-TGGCTATGTGCTTGGCGTTC-3′;GAPDH, forward, 5′-CGTGTTCCTACCCCCAATGT-3′ and reverse, 5′-AGCCCAAGATGCCCTTCAGT-3′;The results were analyzed using the 2^-ΔΔCT^ method for relative quantification

### Immunohistochemistry staining and scoring

Mouse liver cancer tissues were stained using CS and SIRT5 antibodies. Paraffin-embedded liver tissue sections from each group of mice were subjected to heat-induced antigen retrieval, endogenous enzyme inactivation, and blocking. Afterward, CS and SIRT5 antibodies were applied and incubated for 1 hour. Following PBS washes, secondary antibodies were added and incubated for 30 minutes. After additional PBS washes, the sections were developed with DAB, counterstained with hematoxylin, differentiated in ethanol hydrochloride, dehydrated, mounted, and observed. Immunohistochemical results were analyzed using Image Pro Plus 6.0 software, with protein expression levels represented by the average optical density values.

### Western blotting

Liver tissue (0.1 g) from mice was homogenized in 1 mL RIPA lysis buffer and incubated on ice for 30 minutes. After centrifugation at 12,000 rpm for 20 minutes at 4°C, the supernatant was collected. Protein concentrations from each group were measured using a BCA protein assay kit. Protein samples were mixed with loading buffer and heated at 95°C for 5 minutes to ensure complete denaturation. Fifty micrograms of protein were loaded and separated by SDS-PAGE electrophoresis, then transferred to a PVDF membrane. The membrane was blocked with 5% non-fat dry milk at room temperature for 1 hour, followed by incubation with primary antibodies overnight at 4°C. After three 15-minute washes with TBST, the membrane was incubated with appropriate secondary antibodies at room temperature for 1 hour. Finally, protein signals were detected using an ECL chemiluminescent detection system. GAPDH expression was used as an internal reference to detect the relative expression of target proteins. Antibodies used were as follows: SIRT5 (SantaCruz; sc-271635; 1/1000), CS (SantaCruz; sc-390693; 1/1000), AntiSuccinyllysine Mouse mAb (PTM Biolabs, PTM0419; 1/1000), glyceraldehyde 3 -phosphate dehydrogenase (GAPDH) (Bioss; bs-10900R; 1/10,000); Rabbit Anti-Bax antibody (Bioss; bs-0127R; 1/1000); Rabbit Anti-Caspase-3 antibody (Bioss; bs-0081R; 1 1/1000); Bcl2 Monoclonal antibody (proteintech; 66799-1-Ig; 1/1000).

### Cell transfection

Mutations were introduced at the K375 site of CS to simulate succinylation and de-succinylation by substituting arginine (R) and glutamic acid (E), respectively (Zebrafish Biotech Co.,Ltd,Nanjing,China). The mutated plasmids were then transfected into HepG2 cells. Cells (5 × 10^5 cells/well) were seeded in 6-well plates a few days prior to transfection. Once the cells reached 60-80% confluence, transfection was performed using Lipofectamine 3000 (Thermo Fisher Scientific Waltham, MA, USA).

### Co-immunoprecipitation assay

Co-IP was performed to examine the interaction between SIRT5 and CS in cells. After washing the cells twice with ice-cold PBS, they were lysed in Radio Immunoprecipitation Assay (RIPA) buffer containing a proteinase inhibitor for 30 minutes on ice. The cell lysate was then centrifuged at 12,000 g for 10 minutes at 4°C, and the supernatant was collected. An aliquot of 10 µL was retained as the input control. The remaining supernatant was incubated with SIRT5, CS, or IgG antibodies (2 µg) along with Protein G Plus-Agarose Immunoprecipitation reagent (YJ201, Epizyme Biomedical Technology Co., Ltd,Shanghai,China) overnight at 4°C. IgG was used as a negative control. After incubation, the immunocomplexes were washed three times with lysis buffer. The immunoprecipitated proteins were eluted by boiling in 1× loading buffer at 100°C for 10 minutes. The resulting protein-protein complexes were analyzed by Western blotting. Protein signals were detected and quantified using the Tanon 5200 system (Tanon Science & Technology Co.,Ltd,Shanghai, China). Antibodies used were as follows: goat anti-rabbit IgG (Proteintech; 30000-0-AP; 1/500).

### Mito-tracker red CMXRos staining

To measure the level of biologically active mitochondria in cells, the cells were incubated with 200 nM Mito-Tracker Red CMXRos working solution (C1035, Beyotime Biotechnology Co.,Ltd, Shanghai,China) at 37°C in the dark for 20 minutes. After incubation, the cells were washed three times with warm PBS. The stained cells were then observed under a Spectral laser scanning confocal microscopy system to visualize the mitochondrial distribution and activity(FV10i,Olympus Corporation,Tokyo, Japan).

### Measurement of intracellular reactive oxygen species

To measure the intracellular ROS levels, the cells were seeded in a 6-well plate and cultured for 24 hours. Then, H2DCF-DA (10 μM) was added to the cells, and the cells were incubated at 37°C for 20 minutes according to the manufacturer’s instructions (S0033, Beyotime Biotechnology Co.,Ltd, Shanghai,China). After incubation, the cells were observed and analyzed using an inverted fluorescence microscope(Axio Oberser A1,Carl Zeiss,Germany) to detect ROS levels.

### EDU-based cell proliferation assay

Cell proliferation was assessed using the Meilun EdU Cell Proliferation Kit with Alexa Fluor 488 (MA0424, Meilun Biotechnology Co., Ltd,Dalian,China). Briefly, the cells were treated as instructed and incubated with 50 μM EdU for 2 hours. After incubation, the cells were fixed and permeabilized. EdU staining was then performed using the EdU reaction solution. Cell nuclei were stained with Hoechst 33342. Finally, images were captured using an inverted fluorescence microscope (Axio Oberser A1,Carl Zeiss,Germany)to assess cell proliferation.

### Colony formation assay

For the colony formation assay, 500 cells were seeded in a 6-well plate and cultured at 37°C for 2 weeks. Afterward, the colonies were fixed with 4% paraformaldehyde and stained with 0.1% crystal violet for 10 minutes. The number of colonies was then counted.

### Citrate synthase activity

Cells were seeded into a 6-well plate and incubated at 37°C for 24 hours. After incubation, mitochondria were isolated from the cells to measure citrate synthase activity using a citrate synthase activity assay kit (BC1060,Solarbio Science&Technology co.,Ltd,Beijing,China)following the manufacturer’s protocol.

### Measurement of cellular ATP levels

Cells were seeded in a 96-well plate and incubated for 24 hours. The ATP content was measured using an ATP assay kit (S0026, Beyotime Biotechnology Co.,Ltd, Shanghai,China) according to the manufacturer’s instructions. The ATP levels in the cells were quantified by spectrophotometry(SpectraMaxi3,Molecular Devices,Austria)

### TUNEL assay for apoptosis detection

Cells (80-90% confluence) were seeded into a 12-well plate and incubated at 37°C for 24 hours. After incubation, the cells were washed twice with PBS. The control and treated groups were fixed with 4% paraformaldehyde for 30 minutes, followed by permeabilization with 0.3% Triton X-100 for 15 minutes. The cells were then stained with TUNEL reagent for 60 minutes and DAPI for 10 minutes to stain the nuclei. Finally, the cells were washed twice with PBS. Tunel Cell Apoptosis Detection Kit(G1502,Servicebio Technology CO.,LTD,Wuhan,China)The apoptotic cells were observed under a fluorescence microscope(Axio Oberser A1,Carl Zeiss,Germany).

### Flow cytometry

Mitochondrial Membrane Potential Assay: The JC-1 dye(G1515,Servicebio Technology CO.,LTD,Wuhan,China) was used to stain the mitochondrial transmembrane potential, and flow cytometry was employed to analyze the stained cells.

Measurement of Intracellular ROS: Following the manufacturer’s instructions, intracellular ROS levels were measured using a reactive oxygen species detection kit (S0033, Beyotime Biotechnology Co.,Ltd, Shanghai,China). Briefly, cells were seeded in a 6-well plate and incubated for 24 hours. Then, H2DCF-DA (10 μM) was added, and the cells were incubated at 37°C for 20 minutes. Finally, flow cytometry was used to analyze the stained cells.

Cell Apoptosis Analysis: Cell apoptosis was detected using the Annexin V-FITC apoptosis detection kit (AF2020, LABLEAD,Beijing,China) according to the manufacturer’s standard protocol. Briefly, cells were collected and resuspended in binding buffer, followed by incubation with Annexin V-FITC for 15 minutes. Apoptosis was analyzed by flow cytometry(BD FACSVerse,BD Biosciences Franklin Lakes, NJ, USA), detecting the different fluorescence signals from the cells.

### Statistical analysis

Data analysis was performed using Prism 8 (GraphPad Software). A p-value of <0.05 was considered statistically significant. The normality of the data was assessed using the Shapiro-Wilk test. The homogeneity of variances between two groups was tested using the F-test, while the Brown-Forsythe test was used to assess the homogeneity of variances across multiple groups. Statistical differences between two groups were analyzed using the Student’s t-test, while comparisons among multiple groups were performed using one-way analysis of variance (ANOVA) followed by t-tests. Data are presented as mean ± standard deviation (SD). For non-normally distributed data, the non-parametric Kruskal-Wallis rank-sum test was used.

## Results

### Succinylation proteomics reveals high succinylation levels of CS in HCC tissues

**Figure 1:**
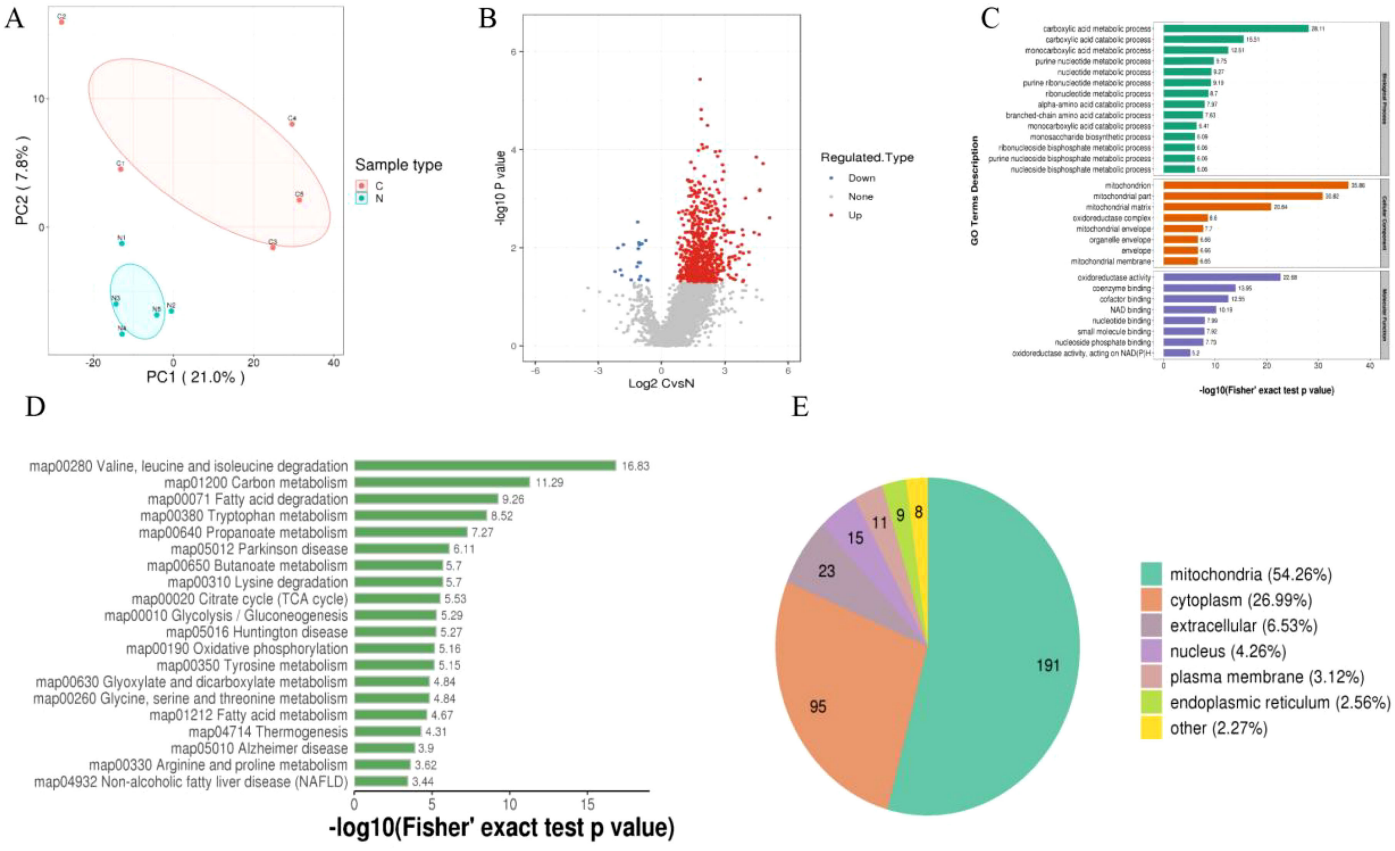
**(A)** PCA analysis of differentially modified proteins, with red representing tumor tissue and blue representing adjacent non-tumor tissue. **(B)** Volcano plot of differentially modified proteins. **(C)** GO enrichment analysis of differentially modified proteins. **(D)** KEGG enrichment analysis of differentially modified proteins. **(E)** Subcellular localization enrichment of differentially modified proteins.

Using 4D-Label-free technology, we analyzed the proteins in tumor and adjacent tissues from five HCC patients. A total of 787,211 secondary spectra were obtained through mass spectrometry. After searching the spectra against theoretical protein databases, 104,359 valid spectra were identified. From these spectra, 18,441 peptides were identified, of which 8,542 were modified peptides. In total, 1,962 proteins were identified. PCA analysis of the proteins from tumor and adjacent tissues was performed, and the results revealed that the PCA explained 28.8% of the total variance between the two groups. PC1 and PC2 accounted for 21% and 7.8% of the overall variation, respectively. A clear separation trend was observed between the differentially modified proteins in the two groups, indicating a significant difference in the succinylation-modified proteins between tumor and adjacent tissues (**Figure 1A**).

Next, we set a threshold of P<0.05 and Log FC > 1.5 for significantly upregulated modifications, and Log FC < -1.5 for significantly downregulated modifications. As a result, we identified 358 differentially modified proteins, including 342 upregulated and 16 downregulated proteins (**Figure 1B**). To gain a comprehensive understanding of the differentially modified proteins identified in the dataset, we performed detailed annotations from various perspectives, including Gene Ontology (GO), KEGG pathways, and subcellular localization, to better understand the functions and characteristics of these proteins. The GO annotation was categorized into three main classes: Biological Process (BP), Cellular Component (CC), and Molecular Function (MF), each providing insights into the biological roles of these differentially modified proteins. The GO analysis showed that the differentially modified proteins were primarily enriched in the following categories: in BP, they were mainly associated with the carboxylic acid metabolic process, carboxylic acid catabolic process, and monocarboxylic acid metabolic process; in CC, they were mainly enriched in the mitochondrion, mitochondrial part, and mitochondrial matrix; and in MF, they were predominantly involved in oxidoreductase activity, coenzyme binding, and cofactor binding (**Figure 1C**). The KEGG analysis revealed that the differentially modified proteins were predominantly enriched in pathways related to valine, leucine, and isoleucine degradation, carbon metabolism, and fatty acid degradation (**Figure 1D**). Additionally, subcellular localization analysis of the differentially modified proteins showed that they were primarily localized in the mitochondria, cytoplasm, extracellular space, nucleus, plasma membrane, and endoplasmic reticulum (**Figure 1E**).

**Figure 2:**
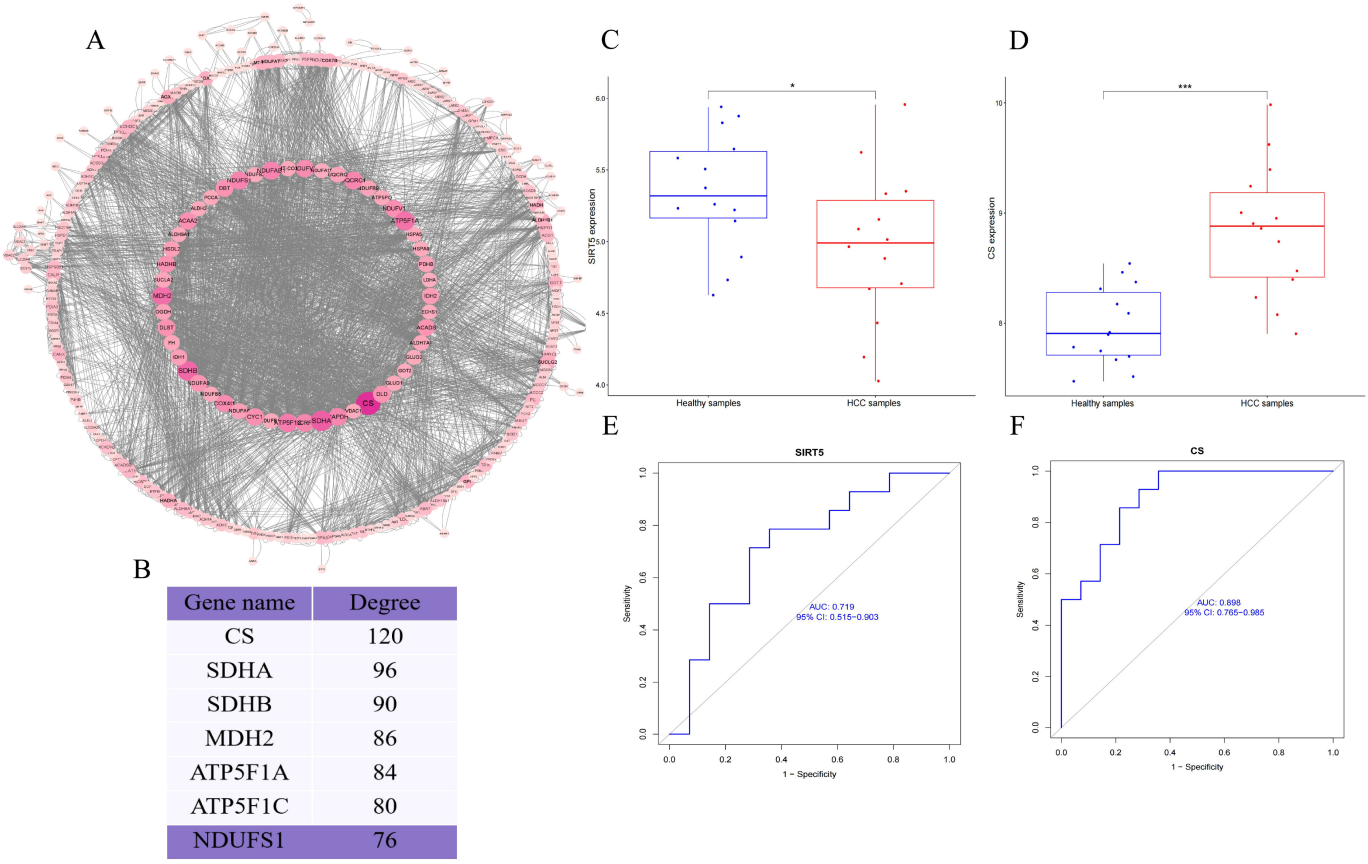
**(A)** PPI network of differentially modified proteins; **(B)** Importance scores of key proteins in the PPI network; **(C, D)** Box plots of the differential expression of CS and SIRT5; **(E, F)** AUC curves and confidence intervals for CS and SIRT5 expression. *P* < 0.05 (*), and *P* < 0.001 (***).

Finally, we retrieved the differential modification protein interaction relationships by comparing the database IDs or protein sequences with the STRING (v.10.5) protein-protein interaction database. A confidence score > 0.7 (high confidence) was set as the threshold for extracting the interactions. The resulting protein-protein interaction network of differentially modified proteins was then visualized using Cytoscape software. After visualization, we used the CytoNCA plugin to calculate the centrality of each protein in the PPI network based on degree centrality. The size and color depth of the protein nodes were determined according to the centrality values, with higher centrality corresponding to larger node size and darker color intensity (**Figure 2A**). The CytoNCA results indicated that Citrate Synthase (CS) had the highest score in the PPI network. We hypothesize that the succinylation of CS may play a crucial role in the initiation and progression of HCC (**Figure 2B**). After reviewing relevant literature, we learned that CS can be targeted by other host factors, which regulate its function by inducing modifications. Additionally, we discovered that some studies have confirmed that CS serves as a substrate for NAD-Dependent Protein Deacylase Sirtuin-5 (SIRT5), and SIRT5 can induce the desuccinylation of CS.

To further contextualize our experimental findings, we analyzed expression data from the GSE84402 dataset. CS was significantly upregulated and SIRT5 markedly downregulated in HCC tissues compared to normal controls (**Figures 2C, D**). Although transcript-level data do not directly reflect protein activity or post-translational modification, these results support the pathological relevance of CS/SIRT5 dysregulation in HCC. Moreover, ROC analysis demonstrated that the expression of CS and SIRT5 could distinguish HCC from normal tissue with high accuracy (**Figures 2E, F**), thereby reinforcing the rationale for exploring their mechanistic interplay in mitochondrial metabolism and apoptosis regulation in liver cancer cells.

### Differential expression of SIRT5 and CS in normal and HCC samples

**Figure 3:**
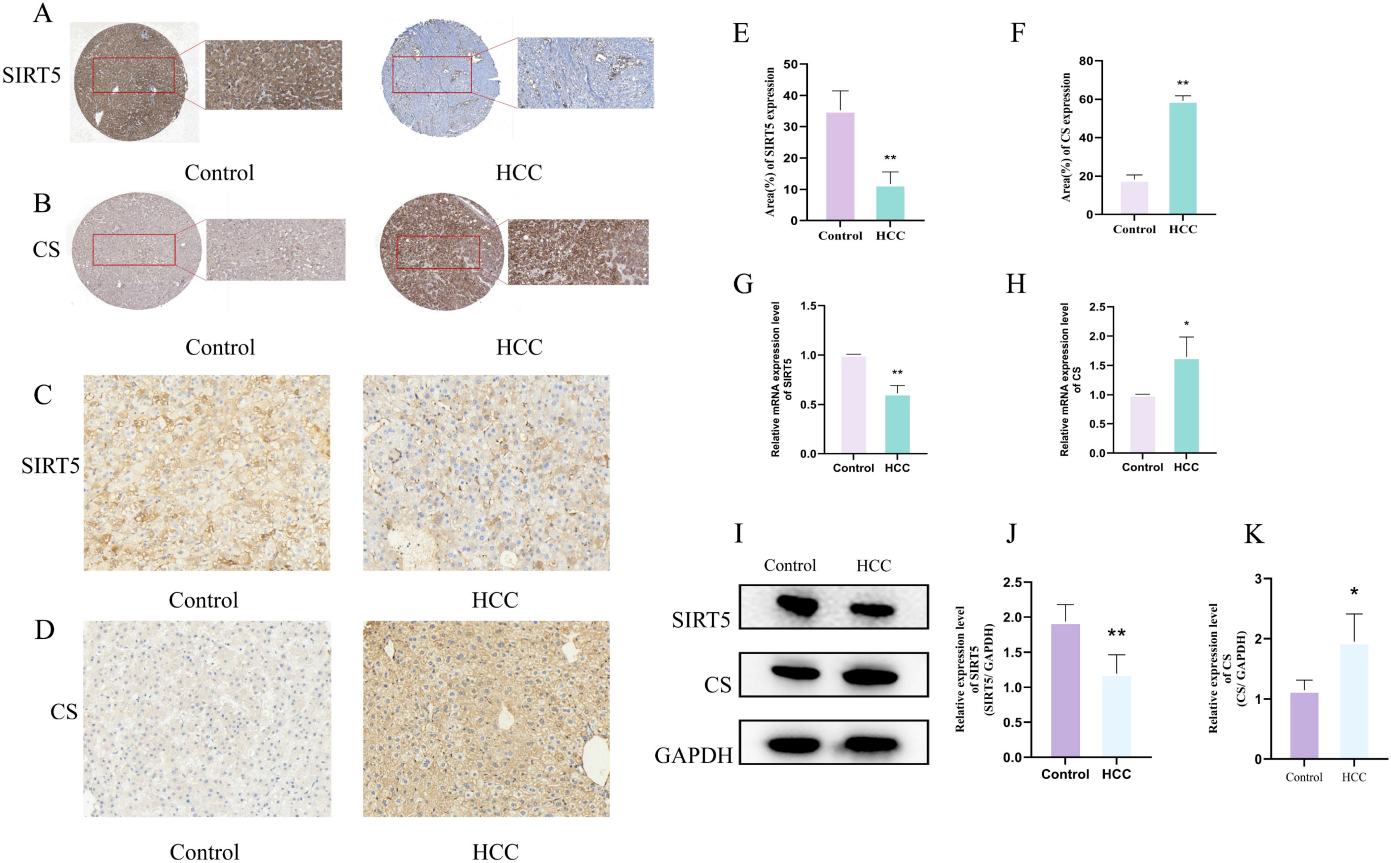
**(A, B)** Expression of SIRT5 **(A)** and CS **(B)** in pathological tissue sections from the HPA database. **(C, D)** IHC staining of SIRT5 **(C)** and CS **(D)** in normal and HCC mouse liver tissues (n=3). **(E, F)** Bar plots showing the differences in positive expression of SIRT5 and CS in normal and HCC mouse liver tissues (n=3). **(G, H)** Bar plots showing the differential mRNA expression of CS and SIRT5 in normal and HCC mouse liver tissues (n=3). **(I)** Protein expression of CS and SIRT5 in normal and HCC mouse liver tissues. **(J, K)** Bar plots showing the differential protein expression of CS and SIRT5 in normal and HCC mouse liver tissues (n=3). *P* < 0.05 (*), *P* < 0.01 (**).

We further explored the expression of SIRT5 and CS in human HCC and adjacent tissues using the HPA database. Immunohistochemical (IHC) results revealed that, compared to adjacent tissues, the expression of SIRT5 was significantly lower in HCC tissues, whereas the expression of CS was significantly higher (**Figures 3A, B**). To enhance the reliability of our findings, we also conducted IHC (**Figures 3C, D**), RT-qPCR (**Figures 3E, F**), and Western blotting (WB) (**Figures 3I–K**) experiments in normal and HCC mouse liver tissues. The results showed that SIRT5 expression was significantly reduced (P<0.05) in HCC mouse liver tissues compared to normal liver tissues, while CS expression was significantly increased (P<0.05) in HCC mouse liver tissues. These findings in mouse liver tissues were consistent with those observed in the HPA data for human samples.

### SIRT5 induces de-succinylation of CS

**Figure 4:**
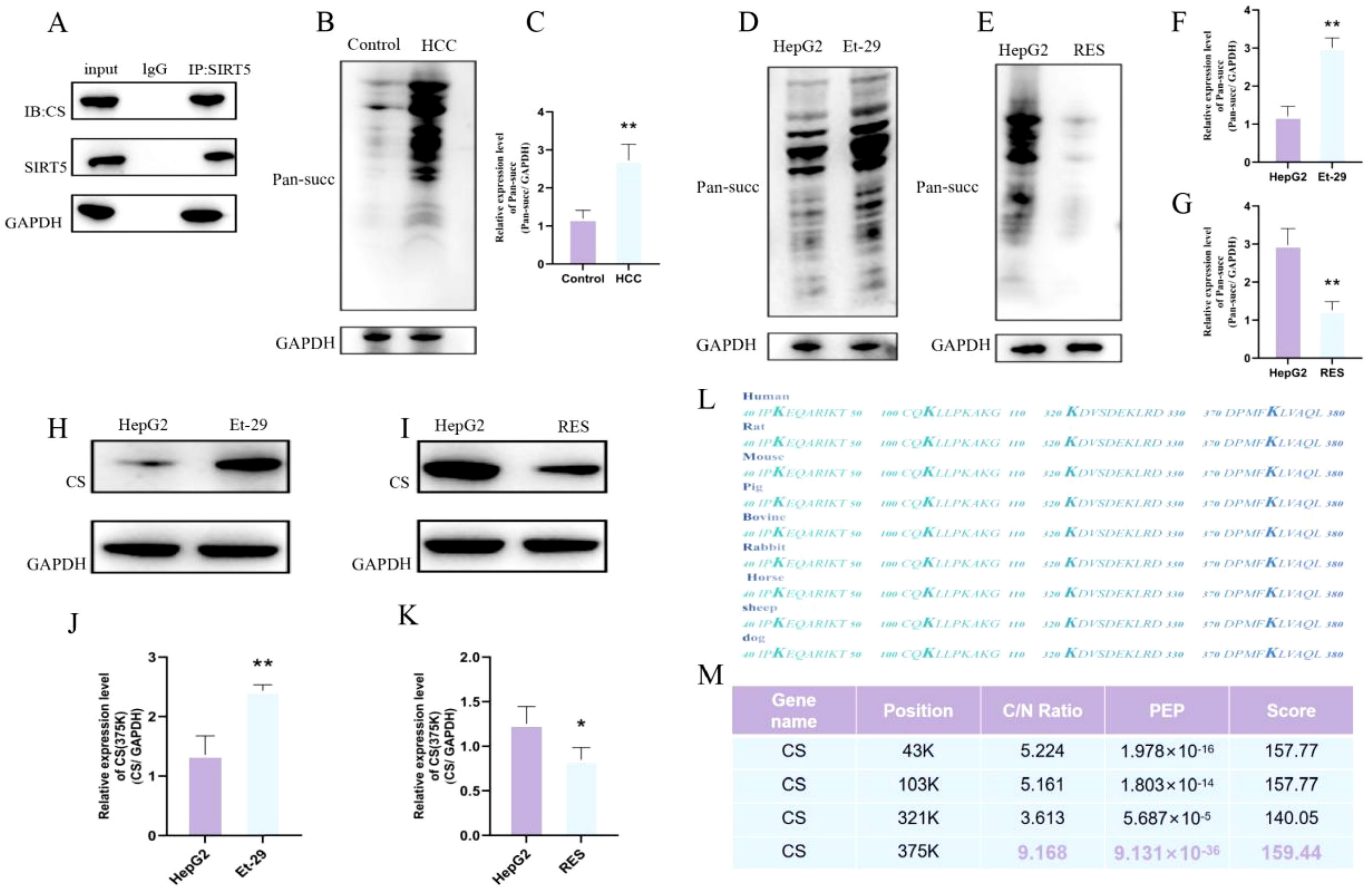
**(A)** Co-immunoprecipitation of CS and SIRT5. **(B, C)** Succinylation expression levels and differential expression bar graphs in normal mouse liver and HCC mouse liver tissues. **(D, F)** Succinylation level differences between HepG2 cells and HepG2 cells treated with Et-29 (n=3). **(E, G)** Succinylation level differences between HepG2 cells and HepG2 cells treated with RES (n=3). **(H, J)** Differential succinylation levels of CS in HepG2 cells and HepG2 cells treated with Et-29 (n=3). **(I, K)** Differential succinylation levels of CS in HepG2 cells and HepG2 cells treated with RES (n=3). **(L)** Comparison of succinylation modification sites on CS across different mammals. **(M)** Ratio, PEP, and Score of succinylation modification sites on CS. *P* < 0.05 (*), *P* < 0.01 (**).

To determine whether there is an interaction between SIRT5 and CS, as well as the nature of this interaction, we first performed immunoprecipitation (Co-IP) assays. The results confirmed a significant interaction between SIRT5 and CS (**Figure 4A**). Next, we performed succinylation detection on the liver tissues of both normal and HCC mice. The results showed a significant increase in succinylation levels in the liver tissues of HCC mice (**Figures 4B, C**). Subsequently, we treated HepG2 cells with either the SIRT5 inhibitor (Et-29) or the SIRT5 activator, Resveratrol (RES), and assessed the succinylation levels in both groups of cells using an anti-succinylation pan-antibody. The results revealed that after the addition of the SIRT5 inhibitor, the succinylation levels in HepG2 cells were significantly elevated, while treatment with the SIRT5 activator resulted in a significant decrease in succinylation levels. These findings demonstrate that the inhibition/activation of SIRT5 can significantly modulate the overall succinylation levels in HepG2 cells (**Figures 4D–G**). Finally, we assessed the succinylation levels of CS in HepG2 cells treated with either the SIRT5 inhibitor or the SIRT5 activator. The results showed that when SIRT5 was inhibited, the succinylation levels of CS were significantly elevated. In contrast, upon SIRT5 activation, the succinylation levels of CS were significantly reduced(**Figures 4H–K**). These findings confirm that the succinylation levels of CS are directly regulated by SIRT5. In our previous post-translational modification proteomics analysis, we observed that the succinylation levels of CS were significantly elevated in tumor tissues from HCC patients. Additionally, we identified four specific lysine sites on CS that undergo succinylation, namely K43, K103, K321, and K375. After comparing the CS sites across various mammalian species, we found that the four succinylation sites on CS, namely K43, K103, K321, and K375, are highly conserved in mammals. Upon further quantitative analysis of these four CS succinylation sites, we discovered that the succinylation level at the K375 site exhibited the most significant differential expression between tumor and adjacent normal tissues in HCC patients(**Figures 4L, M**). Therefore, we hypothesize that the K375 site of CS may be the most critical site for succinylation modification in HCC.

### Succinylation of the K375 site of CS promotes HepG2 cell survival

**Figure 5:**
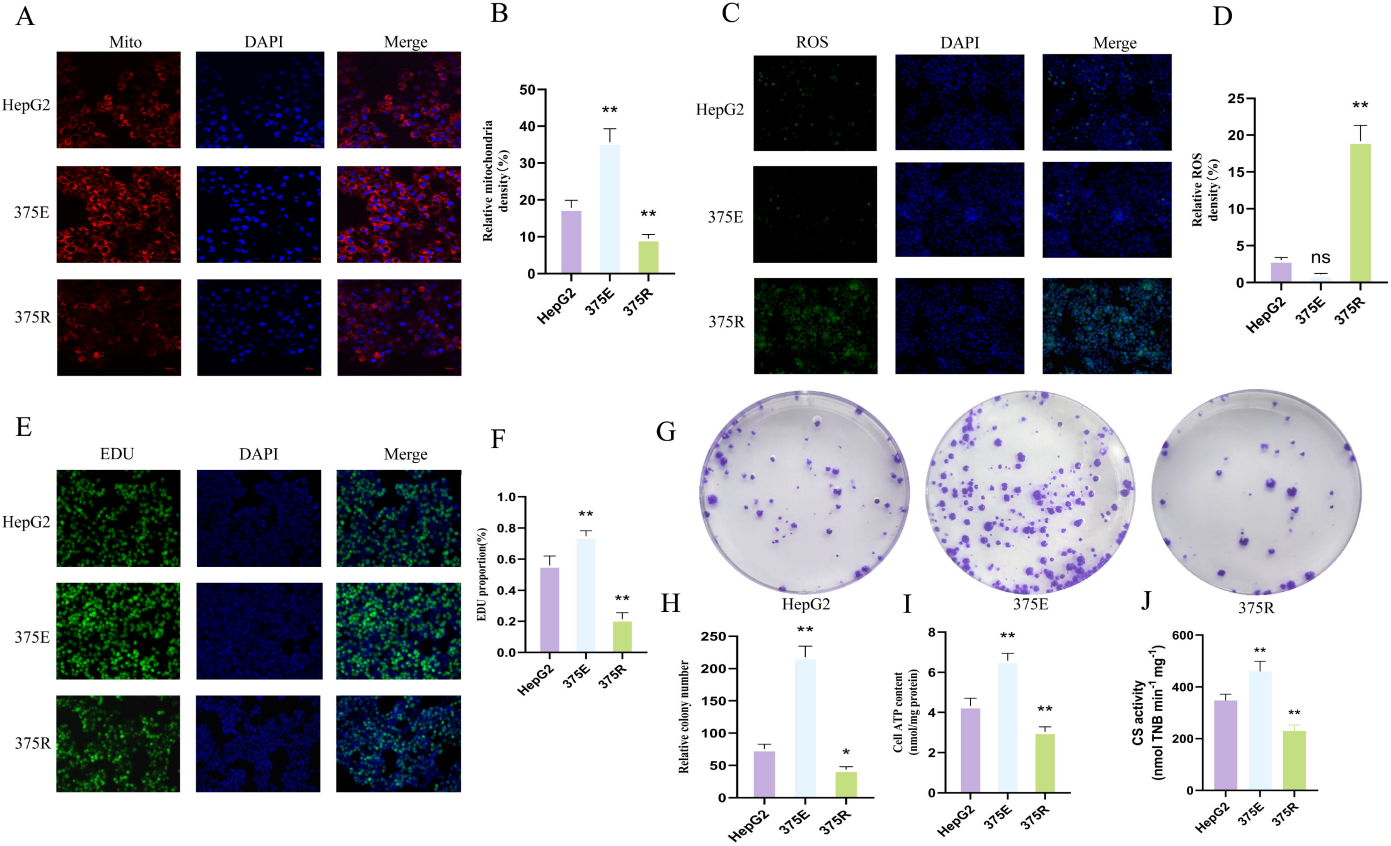
**(A, B)** Comparison of mitochondrial fluorescence intensity in HepG2, CSK375E, and CSK375R treated cells (n=3); **(C, D)** Comparison of ROS fluorescence intensity in HepG2, CSK375E, and CSK375R treated cells (n=3); **(E, F)** Comparison of cell proliferation in HepG2, CSK375E, and CSK375R treated cells (n=3); **(G, I)** Comparison of colony formation in HepG2, CSK375E, and CSK375R treated cells (n=3); **(H)** Comparison of ATP levels in HepG2, CSK375E, and CSK375R treated cells (n=3). **(J)** CS enzyme activities of HepG2, CSK375E, and CSK375R. *P* < 0.05 (*), *P* < 0.01 (**).

To clarify the effect of CS K375 succinylation on tumor cells, we mutated lysine (K) to glutamic acid (E) to mimic succinylation, and K to arginine (R) to mimic dessuccinylation. The mutant plasmids were transfected into HepG2 cells. In our previous succinylation proteomics analysis, we observed that differentially modified proteins were predominantly enriched in the mitochondria. Therefore, we first investigated the effects of CSK375E and CSK375R on the mitochondria in HepG2 cells. The results showed that, compared to normal HepG2 cells, the mitochondrial fluorescence intensity in HepG2 cells transfected with CSK375E was significantly increased. In contrast, the mitochondrial fluorescence intensity in HepG2 cells treated with CSK375R was significantly reduced (**Figures 5A, B**). This confirms that the succinylation of the CSK375 site significantly enhances mitochondrial activity in HepG2 cells. Subsequently, we measured the ROS fluorescence in all three groups of cells. The results showed that the ROS fluorescence intensity in HepG2 cells treated with CSK375E was significantly reduced compared to normal HepG2 cells. Although the difference between the two groups was not statistically significant, the ROS fluorescence intensity in cells treated with CSK375R was significantly increased. These findings suggest that the succinylation of the CSK375 site also regulates the expression of ROS in HepG2 cells (**Figures 5C, D**). Next, we assessed the proliferation levels of the three groups of cells using EdU staining. The results showed that the proliferation of HepG2 cells was significantly increased after treatment with CSK375E, while the proliferation of cells treated with CSK375R was notably reduced (**Figures 5E, F**). Additionally, we measured the ATP content in the three groups of cells, and the results revealed that the ATP levels were significantly higher in the CSK375E-treated cells, whereas ATP levels were significantly lower in the CSK375R-treated cells (**Figure 5I**). Then, we inoculated 200 cells from each of the three groups into a 6-well plate for colony formation assays. After two weeks of cell culture, colonies were stained with crystal violet and counted. The results showed that the number of colonies was significantly increased in the CSK375E-treated HepG2 cells, while the number of colonies was significantly reduced in the CSK375R-treated cells (**Figures 5G, H**).Finally, we measured citrate synthase activity using a commercial assay kit.The results showed that CS enzyme activity was significantly increased after treatment with CSK375E,while the CS enzyme activity was notably reduced after treated with CSK375R (**Figure 5J**). After a series of tests, we conclude that succinylation at the K375 site of CS may enhance the CS enzyme activity, mitochondrial activity and ATP content in HepG2 cells, while also reducing ROS levels, ultimately promoting cell proliferation.

### SIRT5 activation significantly inhibits CS succinylation at site 375 and promotes apoptosis in HepG2 cells

**Figure 6:**
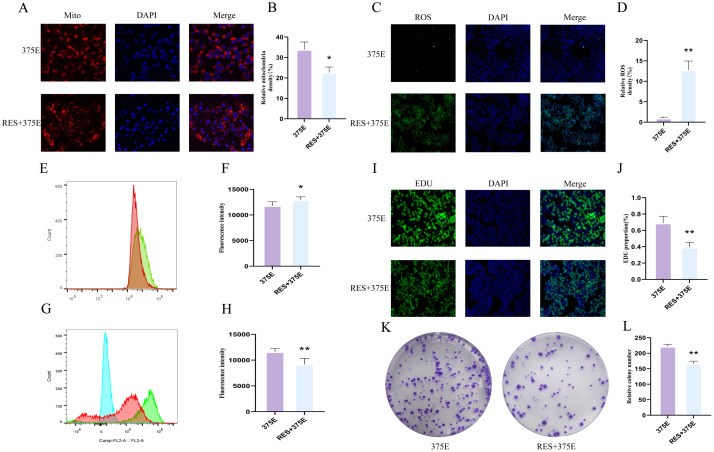
**(A, B)** Comparison of mitochondrial fluorescence intensity between K375E cells and K375E cells treated with RES (n=3); **(C, D)** Comparison of ROS fluorescence intensity between K375E cells and K375E cells treated with RES (n=3); **(E, F)** Comparison of ROS flow cytometry levels between K375E cells and K375E cells treated with RES (n=5); **(G, H)** Comparison of JC1 flow cytometry levels between K375E cells and K375E cells treated with RES (n=5); **(I, J)** Comparison of EDU levels between K375E cells and K375E cells treated with RES (n=3); **(K, L)** Comparison of colony formation numbers between K375E cells and K375E cells treated with RES (n=3). *P* < 0.05 (*), *P* < 0.01 (**).

In our previous study, we have already confirmed that the succinylation modification of the CS K375 site promotes the proliferation of HepG2 cells. Additionally, we found that activation of SIRT5 can significantly inhibit the succinylation of CS at the K375 site. Therefore, we treated K375E HepG2 cells with a SIRT5 activator to determine whether the activation of SIRT5 can reverse the effects of K375E treatment in HepG2 cells. First, we assessed the mitochondrial fluorescence intensity in both groups of cells. After treatment with the SIRT5 activator, the mitochondrial fluorescence intensity in K375E HepG2 cells significantly decreased (**Figures 6A, B**). Conversely, the intracellular ROS fluorescence intensity and levels significantly increased (**Figures 6C–F**). Additionally, we examined the JC1 levels and cell proliferation rates in the two groups. The results showed a significant reduction in JC1 levels in K375E HepG2 cells treated with the SIRT5 activator (**Figures 6G, H**), while the EDU (**Figures 6I, J**) and colony formation (**Figures 6K, L**) assays demonstrated a marked decrease in HepG2 cell proliferation.

**Figure 7:**
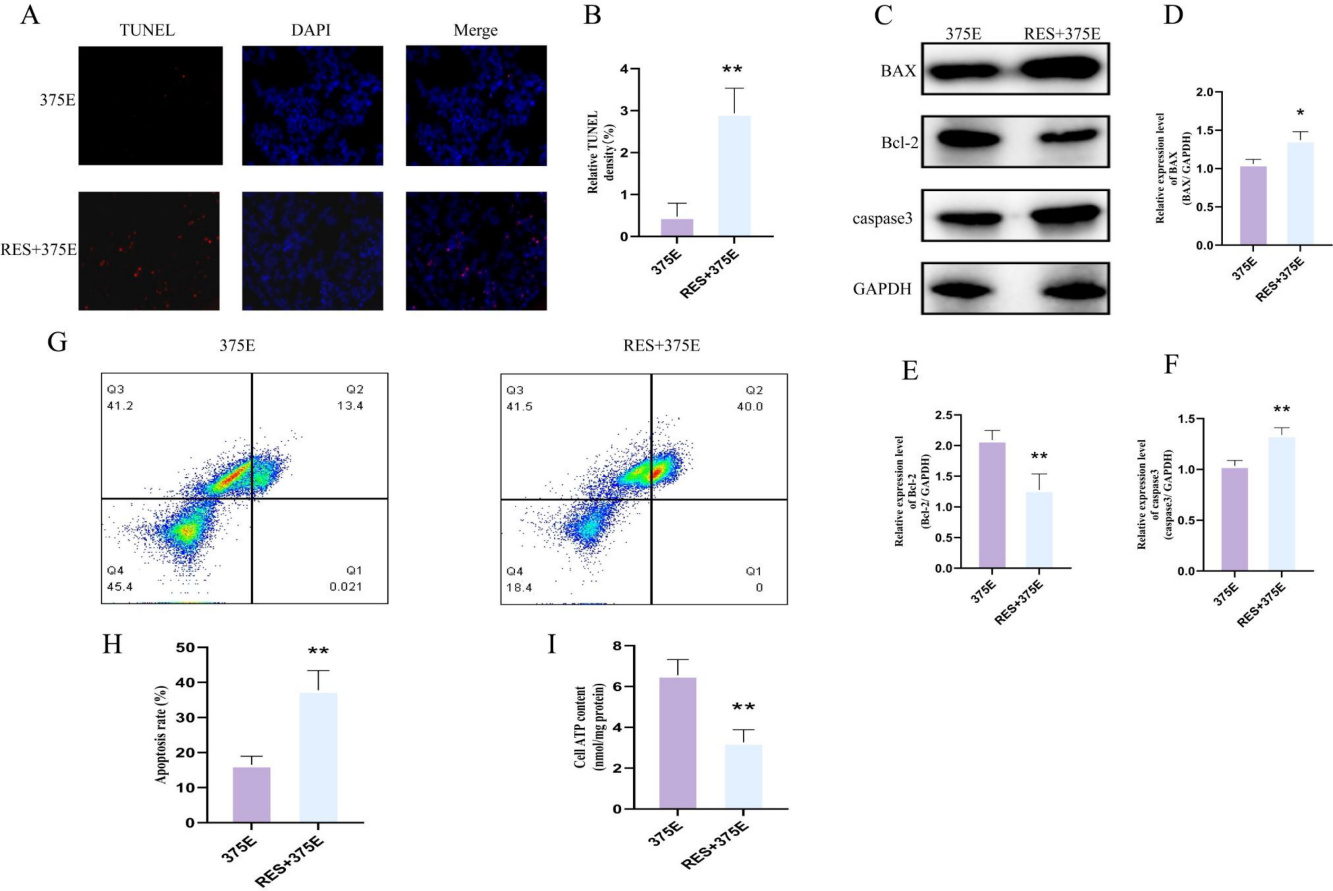
**(A, B)** Comparison of TUNEL fluorescence between K375E cells and K375E cells treated with RES (n=3); **(C–F)** Comparison of BAX, Bcl-2, and caspase3 protein expression levels between K375E cells and K375E cells treated with RES (n=3); **(G, H)** Comparison of apoptosis flow cytometry levels between K375E cells and K375E cells treated with RES (n=5); **(I)** Comparison of ATP levels between K375E cells and K375E cells treated with RES (n=3). *P* < 0.05 (*), *P* < 0.01 (**).

Next, we examined the apoptotic levels and ATP content in both groups of cells. TUNEL fluorescence assays showed that after treatment with the SIRT5 activator, the apoptosis rate of HepG2 cells significantly increased (**Figures 7A, B**). We also performed flow cytometry analysis of apoptosis in both groups (**Figures 7G, H**), and the results were consistent with the trend observed in the TUNEL assay. The ATP results revealed a significant reduction in ATP content in HepG2 cells treated with the SIRT5 activator (**Figure 7I**). Additionally, we used Western blot (WB) to assess the protein expression levels of BAX, Bcl-2, and caspase-3 in both groups. The results showed that the expression levels of BAX and caspase-3 significantly increased after treatment with the SIRT5 activator, while Bcl-2 expression significantly decreased (**Figures 7C–F**). Through the above experimental methods, we found that the succinylation of the CS K375 site can promote HepG2 cell proliferation. However, after treatment with the SIRT5 activator, the proliferation of HepG2 cells induced by K375E was significantly reversed, and apoptosis in HepG2 cells was also promoted.

## Discussion

Lysine succinylation is an evolutionarily conserved PTM that affects the activity, stability, and localization of various proteins, particularly metabolic enzymes (24–26). It plays a key role in regulating numerous biological processes within the cell, such as mitochondrial metabolism, energy production, and oxidative stress responses (27–29). Succinylation has been reported to be closely associated with liver diseases (30).Succinyl-CoA derived from the TCA cycle serves as the primary substrate for succinylation. The accumulation of succinyl-CoA, as well as the knockout of the desuccinylase SIRT5, both induce histone hypersuccinylation (31). In liver cancer tissues, the level of succinylation is higher than in adjacent tissues, and elevated succinylation is associated with poorer prognosis. However, its underlying mechanisms remain largely unclear. The study revealed that SIRT5 deletion in YAP+ hepatocytes upregulated ACOX2 succinylation and activity, thereby promoting HCC immune evasion and growth (22). In addition, OXCT1-mediated LACTB succinylation inactivated this tumor suppressor, which accelerated hepatocarcinogenesis (32).Given the complex role of succinylation signaling in HCC, further studies are warranted to explore the underlying mechanisms by which succinylation contributes to hepatocarcinogenesis and progression. To comprehensively explore lysine succinylation events in HCC, we employed an unbiased, global proteomic strategy rather than targeting a specific protein. This enabled us to systematically identify high-confidence and disease-relevant modification sites and regulatory enzymes. In this study, we found that the expression of the de-succinylase SIRT5 is reduced in human liver cancer tissues, which promotes lysine succinylation in HCC. CS exhibits high levels of succinylation, and its expression is significantly increased in tumor tissues. Importantly, we discovered that high succinylation at the K370 site of CS enhances mitochondrial metabolism and promotes the proliferation of liver cancer cells. These findings suggest that CS succinylation may contribute to the progression of HCC.

CS is a crucial mitochondrial enzyme located in the nuclear DNA, synthesized in the cytoplasmic ribosomes and subsequently transferred to the mitochondrial matrix, where it catalyzes the conversion of acetyl-CoA and oxaloacetate into citrate. CS serves as a key node linking glycolysis and the TCA cycle (33), determining the flux of the entire TCA cycle and the energy generated by intracellular metabolic processes (34). Additionally, CS is a quantitative marker for mitochondrial integrity, function, quality, and mitochondrial respiratory chain enzymes (35, 36).

The role of CS in the development of cancer remains insufficiently explored, and CS may play a dual role in tumorigenesis. In ovarian cancer, the expression of CS is upregulated, promoting cell proliferation, invasion, and migration (37). In cervical cancer cells, inhibition of citrate synthase expression induces epithelial-mesenchymal transition (EMT), thereby enhancing the malignant characteristics of tumor cells (38). This study found that the level of succinylation of CS is elevated in human liver cancer tissues, and the expression of CS protein is significantly increased. However, the mechanisms regulating CS post-translational modifications and its functional role in liver cancer progression remain unclear. Research indicates that succinyltransferases and de-succinylases promote or inhibit the progression of various cancers by regulating the succinylation levels of substrate targets (39–41).SIRT5, the only known mitochondrial desuccinylase, is associated with metabolic disorders and cancer (42, 43). Park’s work showed that SIRT5 is a central regulator of Lys succinylation in mammalian cells, they identified SIRT5 preferentially targets enzymes involved in the TCA cycle and fatty acid metabolism. Notably, critical metabolic enzymes including CS, SDHA, and IDH2 were found to be regulated by SIRT5-mediated desuccinylation (16).Further investigation by Rardin. et al. identified specific lysine succinylation sites on CS that are regulated by SIRT5 (44).In addition, SIRT5 deficiency has been associated with enhanced succinylation of CS in both colorectal tumor model and subarachnoid hemorrhage model (23, 45).These findings demonstrate that SIRT5 directly interacts with CS, and its downregulation results in hyper-succinylation of CS. In this study, we found that the expression of SIRT5 protein is decreased in human liver cancer tissues and HCC mice, and the reduced expression of SIRT5 is associated with elevated succinylation levels of CS. Furthermore, we observed that the inhibition or activation of SIRT5 significantly regulates the overall succinylation levels of CS in HepG2 cells. This study suggests that SIRT5-mediated regulation of CS succinylation may serve as a novel therapeutic target for hepatocellular carcinoma.

LC-MS/MS analysis revealed that a decrease in SIRT5 expression leads to a significant increase in the succinylation of four lysine residues (K43, K103, K321, K375) in CS, and these modifications are highly conserved across different species. We found that the succinylation level of the K375 residue in CS exhibited the most significant expression difference between tumor tissues and adjacent normal tissues in patients with HCC. Therefore, we utilized CS K375E and K375R mutants to simulate succinylation and desuccinylation of CS, respectively, to further investigate the mechanism by which CS succinylation affects the progression of hepatocellular carcinoma. Prior research indicating that succinylation may influence protein conformation and substrate-binding efficiency by introducing localized negative charges (16). In this study, we found that the CS K346R mutant, which simulates the desuccinylation of CS, resulted in decreased CS enzyme activity, significantly reduced mitochondrial activity and ATP levels, increased ROS levels, and markedly inhibited the proliferation of liver cancer cells. In contrast, succinylation at the K375 site promoted the proliferation of HepG2 cells. In summary, this finding suggests that SIRT5-mediated de-succinylation of K375 in CS, through reducing CS expression, leads to impaired TCA cycle function, disrupted mitochondrial energy metabolism, decreased membrane potential, and triggered mitochondrial oxidative stress, ultimately resulting in apoptosis of HepG2 cells.

Previous studies have demonstrated that various Sirtuin family members contribute to metabolic reprogramming in hepatocellular carcinoma through distinct mechanisms. While SIRT1, SIRT3, and SIRT6 regulate glycolysis, lipid metabolism, and epigenetic remodeling via acetylation and deacetylation (46–48), SIRT5 is uniquely localized in mitochondria and governs energy-rich acyl modifications such as succinylation and glutarylation. Unlike acetylation, which often alters protein-protein interactions or nuclear localization, succinylation can introduce larger structural and electrostatic changes, more profoundly impacting enzyme catalysis (49). In this study, we focus on SIRT5-mediated desuccinylation of CS, revealing a novel regulatory layer of mitochondrial metabolic adaptation in HCC. Our findings suggest that succinylation of CS at K375 may represent a precise regulatory mechanism of mitochondrial function and a potential target for HCC metabolic intervention.

However, this study encompasses several limitations that merit careful consideration. First, although the role of SIRT5-mediated CS desuccinylation has been elucidated through *in vitro* experiments and mouse models, its validation within large-scale clinical HCC cohorts remains to be conducted. Second, while site-directed mutations at K375 (K375E/K375R) effectively mimic succinylation states, their physiological relevance necessitates further substantiation via knock-in mouse models or patient-derived xenograft (PDX) systems. Third, our investigation predominantly concentrated on the metabolic ramifications of CS succinylation within mitochondria, leaving unexplored its potential contributions to tumor microenvironment remodeling, immune regulation, and therapeutic resistance. Future studies will comprehensively delineate the mechanistic and translational implications of CS succinylation in hepatocellular carcinoma pathogenesis.

## Conclusion

In summary, Overall, we discovered through succinylome profiling that there is a significant difference in the succinylation of CS between liver cancer tissue and adjacent tissue. Additionally, both *in vivo* and *in vitro* experiments confirmed that high succinylation of CS can enhance mitochondrial activity, thereby promoting the proliferation of liver cancer cells. Finally, we also found that SIRT5 can reverse the high succinylation of CS and induce apoptosis in liver cancer cells, exerting an anti-tumor effect.

## Data Availability

The datasets presented in this study can be found in online repositories. The names of the repository/repositories and accession number(s) can be found in the article/supplementary material.

## References

[1] Bray F Laversanne M Sung H Ferlay J Siegel RL Soerjomataram I. Global cancer statistics 2022: GLOBOCAN estimates of incidence and mortality worldwide for 36 cancers in 185 countries. CA Cancer J Clin. (2024) 74:229–63. doi: 10.3322/caac.21834 38572751

[2] Llovet JM Kelley RK Villanueva A Singal AG Pikarsky E Roayaie S. Hepatocellular carcinoma. Nat Rev Dis Primers. (2021) 7:6. doi: 10.1038/s41572-020-00240-3 33479224 PMC13537433

[3] Satriano L Lewinska M Rodrigues PM Banales JM Andersen JB. Metabolic rearrangements in primary liver cancers: cause and consequences. Nat Rev Gastroenterol Hepatol. (2019) 16:748–66. doi: 10.1038/s41575-019-0217-8 31666728

[4] De Matteis S Ragusa A Marisi G De Domenico S Casadei Gardini A Bonafè M. Aberrant metabolism in hepatocellular carcinoma provides diagnostic and therapeutic opportunities. Oxid Med Cell Longev. (2018) 2018:7512159. doi: 10.1155/2018/7512159 30524660 PMC6247426

[5] Hsu PP Sabatini DM. Cancer cell metabolism: Warburg and beyond. Cell. (2008) 134:703–7. doi: 10.1016/j.cell.2008.08.021 18775299

[6] Lu M Wu Y Xia M Zhang Y. The role of metabolic reprogramming in liver cancer and its clinical perspectives. Front Oncol. (2020) 14:1454161. doi: 10.3389/fonc.2024.1454161 PMC11602425 39610917

[7] Arnold PK Finley LWS. Regulation and function of the mammalian tricarboxylic acid cycle. J Biol Chem. (2023) 299:102838. doi: 10.1016/j.jbc.2022.102838 36581208 PMC9871338

[8] Anderson NM Mucka P Kern JG Feng H. The emerging role and targetability of the tca cycle in cancer metabolism. Protein Cell. (2018) 9:216–37. doi: 10.1007/s13238-017-0451-1 PMC5818369 28748451

[9] Du D Liu C Qin M Zhang X Xi T Yuan S. Metabolic dysregulation and emerging therapeutical targets for hepatocellular carcinoma. Acta Pharm Sin B. (2022) 12:558–80. doi: 10.1016/j.apsb.2021.09.019 PMC8897153 35256934

[10] Remington SJ. Structure and mechanism of citrate synthase. Curr Top Cell Regul. (1992) 33:209–29. doi: 10.1016/b978-0-12-152833-1.50017-4 1499334

[11] Zhang J Baddoo M Han C Strong MJ Cvitanovic J Moroz K. Gene network analysis reveals a novel 22-gene signature of carbon metabolism in hepatocellular carcinoma. Oncotarget. (2016) 7:49232–45. doi: 10.18632/oncotarget.10249 PMC5226503 27363021

[12] Zhang Z Tan M Xie Z Dai L Chen Y Zhao Y. Identification of lysine succinylation as a new post-translational modification. Nat Chem Biol. (2011) 7:58–63. doi: 10.1038/nchembio.495 21151122 PMC3065206

[13] Yang Y Tapias V Acosta D Xu H Chen H Bhawal R. Altered succinylation of mitochondrial proteins, app and tau in alzheimer’s disease. Nat Commun. (2022) 13:159. doi: 10.1038/s41467-021-27572-2 35013160 PMC8748865

[14] Wu X Xu M Geng M Chen S Little PJ Xu S. Targeting protein modifications in metabolic diseases: molecular mechanisms and targeted therapies. Signal Transduct Target Ther. (2023) 8:220. doi: 10.1038/s41392-023-01439-y 37244925 PMC10224996

[15] Sun L Zhang H Gao P. Metabolic reprogramming and epigenetic modifications on the path to cancer. Protein Cell. (2022) 13:877–919. doi: 10.1007/s13238-021-00846-7 34050894 PMC9243210

[16] Park J Chen Y Tishkoff DX Peng C Tan M Dai L. SIRT5-mediated lysine desuccinylation impacts diverse metabolic pathways. Mol Cell. (2013) 50:919–30. doi: 10.1016/j.molcel.2013.06.001 PMC3769971 23806337

[17] Hirschey MD Zhao Y. Metabolic regulation by lysine malonylation, succinylation, and glutarylation. Mol Cell Proteomics. (2015) 14:2308–15. doi: 10.1074/mcp.R114.046664 PMC4563717 25717114

[18] Fiorentino F Castiello C Mai A Rotili D. Therapeutic potential and activity modulation of the protein lysine deacylase sirtuin 5. J Med Chem. (2022) 65:9580–606. doi: 10.1021/acs.jmedchem.2c00687 PMC9340778 35802779

[19] Chen XF Tian MX Sun RQ Zhang ML Zhou LS Jin L. SIRT5 inhibits peroxisomal ACOX1 to prevent oxidative damage and is downregulated in liver cancer. EMBO Rep. (2018) 19(5):e45124. doi: 10.15252/embr.201745124 29491006 PMC5934778

[20] Yihan L Xiaojing W Ao L Chuanjie Z Haofei W Yan S. SIRT5 functions as a tumor suppressor in renal cell carcinoma by reversing the warburg effect. J Transl Med. (2021) 19:521. doi: 10.1186/s12967-021-03178-6 34930316 PMC8690424

[21] Abril YLN Fernandez IR Hong JY Chiang YL Kutateladze DA Zhao Q. Pharmacological and genetic perturbation establish SIRT5 as a promising target in breast cancer. Oncogene. (2021) 40:1644–58. doi: 10.1038/s41388-020-01637-w PMC7935767 33479498

[22] Sun R Zhang Z Bao R Guo X Gu Y Yang W. Loss of SIRT5 promotes bile acid-induced immunosuppressive microenvironment and hepatocarcinogenesis. J Hepatol. (2022) 77:453–66. doi: 10.1016/j.jhep.2022.02.030 35292350

[23] Ren M Yang X Bie J Wang Z Liu M Li Y. Citrate synthase desuccinylation by SIRT5 promotes colon cancer cell proliferation and migration. Biol Chem. (2020) 401:1031–9. doi: 10.1515/hsz-2020-0118 32284438

[24] Yang X Wang Z Li X Liu B Liu M Liu L. SHMT2 desuccinylation by SIRT5 drives cancer cell proliferation. Cancer Res. (2018) 78:372–86. doi: 10.1158/0008-5472.Can-17-1912 29180469

[25] Wang X Shi X Lu H Zhang C Li X Zhang T. Succinylation inhibits the enzymatic hydrolysis of the extracellular matrix protein fibrillin 1 and promotes gastric cancer progression. Adv Sci (Weinh). (2022) 9:e2200546. doi: 10.1002/advs.202200546 35901491 PMC9507347

[26] Qi H Ning X Yu C Ji X Jin Y McNutt MA. Succinylation-dependent mitochondrial translocation of PKM2 promotes cell survival in response to nutritional stress. Cell Death Dis. (2019) 10:170. doi: 10.1038/s41419-018-1271-9 30787272 PMC6382874

[27] Yang Y Gibson GE. Succinylation links metabolism to protein functions. Neurochem Res. (2019) 44:2346–59. doi: 10.1007/s11064-019-02780-x PMC6755074 30903449

[28] Liu Z Wang R Wang Y Duan Y Zhan H. Targeting succinylation-mediated metabolic reprogramming as a potential approach for cancer therapy. BioMed Pharmacother. (2023) 168:115713. doi: 10.1016/j.biopha.2023.115713 37852104

[29] Zhang J Han ZQ Wang Y He QY. Alteration of mitochondrial protein succinylation against cellular oxidative stress in cancer. Mil Med Res. (2022) 9:6. doi: 10.1186/s40779-022-00367-2 35115046 PMC8815146

[30] Yu Q Zhang J Li J Song Y Pan J Mei C. Sirtuin 5-mediated desuccinylation of aldh2 alleviates mitochondrial oxidative stress following acetaminophen-induced acute liver injury. Adv Sci (Weinh). (2024) 11:e2402710. doi: 10.1002/advs.202402710 39159058 PMC11497042

[31] Sun L Zhang H Gao P. Metabolic reprogramming and epigenetic modifications on the path to cancer. Protein Cell. (2022) 13:877–919. doi: 10.1007/s13238-021-00846-7 34050894 PMC9243210

[32] Ma W Sun Y Yan R Zhang P Shen S Lu H. OXCT1 functions as a succinyltransferase, contributing to hepatocellular carcinoma via succinylating LACTB. Mol Cell. (2024) 84:538–51.e7. doi: 10.1016/j.molcel.2023.11.042 38176415

[33] Liu X Cooper DE Cluntun AA Warmoes MO Zhao S Reid MA. Acetate production from glucose and coupling to mitochondrial metabolism in mammals. Cell. (2018) 175:502–13.e13. doi: 10.1016/j.cell.2018.08.040 30245009 PMC6173642

[34] Mukherjee A Srere PA Frenkel EP. Studies of the mechanism by which hepatic citrate synthase activity increases in vitamin B12 deprivation. J Biol Chem. (1976) 251:2155–60. doi: 10.1016/S0021-9258(17)33669-4 818082

[35] Alhindi Y Vaanholt LM Al-Tarrah M Gray SR Speakman JR Hambly C. Low citrate synthase activity is associated with glucose intolerance and lipotoxicity. J Nutr Metab. (2019) 2019:8594825. doi: 10.1155/2019/8594825 30944739 PMC6421790

[36] Yamada T Kimura I Ashida Y Tamai K Fusagawa H Tohse N. Larger improvements in fatigue resistance and mitochondrial function with high- than with low-intensity contractions during interval training of mouse skeletal muscle. FASEB J. (2021) 35:e21988. doi: 10.1096/fj.202101204R 34665879

[37] Chen L Liu T Zhou J Wang Y Wang X Di W. Citrate synthase expression affects tumor phenotype and drug resistance in human ovarian carcinoma. PloS One. (2014) 9:e115708. doi: 10.1371/journal.pone.0115708 25545012 PMC4278743

[38] Lin CC Cheng TL Tsai WH Tsai HJ Hu KH Chang HC. Loss of the respiratory enzyme citrate synthase directly links the warburg effect to tumor Malignancy. Sci Rep. (2012) 2:785. doi: 10.1038/srep00785 23139858 PMC3492867

[39] Jiang M Huang Z Chen L Deng T Liu J Wu Y. SIRT5 promote Malignant advancement of chordoma by regulating the desuccinylation of c-myc. BMC Cancer. (2024) 24:386. doi: 10.1186/s12885-024-12140-w 38532359 PMC10967166

[40] Wang C Zhang C Li X Shen J Xu Y Shi H. CPT1A-mediated succinylation of S100a10 increases human gastric cancer invasion. J Cell Mol Med. (2019) 23:293–305. doi: 10.1111/jcmm.13920 30394687 PMC6307794

[41] Wu Y Chen W Miao H Xu T. SIRT7 promotes the proliferation and migration of anaplastic thyroid cancer cells by regulating the desuccinylation of KIF23. BMC Cancer. (2024) 24:210. doi: 10.1186/s12885-024-11965-9 38360598 PMC10870498

[42] Wu M Tan J Cao Z Cai Y Huang Z Chen Z. Sirt5 improves cardiomyocytes fatty acid metabolism and ameliorates cardiac lipotoxicity in diabetic cardiomyopathy via CPT2 de-succinylation. Redox Biol. (2024) 73:103184. doi: 10.1016/j.redox.2024.103184 38718533 PMC11091707

[43] Wang YQ Wang HL Xu J Tan J Fu LN Wang JL. Sirtuin5 contributes to colorectal carcinogenesis by enhancing glutaminolysis in a deglutarylation-dependent manner. Nat Commun. (2018) 9:545. doi: 10.1038/s41467-018-02951-4 29416026 PMC5803207

[44] Rardin MJ He W Nishida Y Newman JC Carrico C Danielson SR. SIRT5 regulates the mitochondrial lysine succinylome and metabolic networks. Cell Metab. (2013) 18:920–33. doi: 10.1016/j.cmet.2013.11.013 PMC4105152 24315375

[45] Lian J Liu W Hu Q Zhang X. Succinylation modification: A potential therapeutic target in stroke. Neural Regener Res. (2024) 19:781–7. doi: 10.4103/1673-5374.382229 PMC10664134 37843212

[46] Yang H Zhu R Zhao X Liu L Zhou Z Zhao L. Sirtuin-mediated deacetylation of hnRNP A1 suppresses glycolysis and growth in hepatocellular carcinoma. Oncogene. (2019) 38:4915–31. doi: 10.1038/s41388-019-0764-z 30858544

[47] Zhang J Ye J Zhu S Han B Liu B. Context-dependent role of SIRT3 in cancer. Trends Pharmacol Sci. (2024) 45:173–90. doi: 10.1016/j.tips.2023.12.005 38242748

[48] Huang J Su J Wang H Chen J Tian Y Zhang J. Discovery of novel PROTAC SIRT6 degraders with potent efficacy against hepatocellular carcinoma. J Med Chem. (2024) 67:17319–49. doi: 10.1021/acs.jmedchem.4c01223 39323022

[49] Bringman-Rodenbarger LR Guo AH Lyssiotis CA Lombard DB. Emerging roles for SIRT5 in metabolism and cancer. Antioxid Redox Signal. (2018) 28:677–90. doi: 10.1089/ars.2017.7264 PMC5824490 28707979

